# Targeted exon capture and NGS to investigate an undefined myopathy reveal *RYR1* variants

**DOI:** 10.1186/1471-2253-14-S1-A15

**Published:** 2014-08-18

**Authors:** Kathryn Stowell, Elaine Langton, Neil Pollock, Anja Schiemann

**Affiliations:** 1 Institute of Fundamental Sciences, Massey University, Palmerston North, 4442, New Zealand; 2 Department of Anesthesiology, Wellington Hospital, Wellington, 6021, New Zealand; 3 Department of Anesthesiology, Palmerston North Hospital, Palmerston North, 4410, New Zealand

## Background

The family under investigation consists of parents and two daughters, one being the proband. The mother and the proband have elongated facial features. The father and second daughter appear normal. The older daughter presented for elective tonsillectomy aged 8 years. She had severe masseter spasm after suxamethonium. The rest of the procedure was carried out under total intravenous anaesthesia. No blood gas analysis could be done, but a creatine kinase next day was significantly elevated (2934). This led to study of both parents. There was no family history of malignant hyperthermia but an undefined myopathy was suspected in mother and daughter. Both mother and father were diagnosed malignant hyperthermia (MH) susceptible by in vitro contracture test (IVCT). This prompted a DNA analysis for variants associated with MH.

## Materials and methods

Standard histochemistry, biochemistry and electron microscopy were carried out on muscle tissue from the mother. DNA from all four family members was analysed by targeted exon capture and next generation sequencing using the Ion Torrent platform. B-lymphoblastoid cells were generated from all family members and assayed for abnormal calcium release.

## Results

The mother and both daughters carry a premature stop codon in ryanodine receptor subtype 1 (*RYR1*) as well an uncharacterized *RYR1* variant inherited from the father. The mother also carries a second uncharacterized *RYR1* variant, not inherited by either daughter. Muscle histology showed two cox-negative fibres suggestive of a mitochondrial disorder but not definitive. Calcium release assays using B-lymphoblastoid cells suggest a hypersensitive RyR1 channel in all four family members.

## Conclusions

The *RYR1* variants identified cannot be definitively associated with susceptibility to MH, although the functional assays in B-lymphoblastoid cells suggest a hypersensitive channel. It is possible that the undefined myopathy is associated with another gene and the MH susceptible result by IVCT is unrelated to this condition. Further analysis of the family is required for a definitive diagnosis.

